# Investigation of P1/HC-Pro-Mediated ABA/Calcium Signaling Responses via Gene Silencing through High- and Low-Throughput RNA-seq Approaches

**DOI:** 10.3390/v13122349

**Published:** 2021-11-23

**Authors:** Yen-Hsin Chiu, Yu-Ling Hung, Hsin-Ping Wang, Wei-Lun Wei, Qian-Wen Shang, Thanh Ha Pham, Chien-Kang Huang, Zhao-Jun Pan, Shih-Shun Lin

**Affiliations:** 1 Institute of Biotechnology, National Taiwan University, Taipei 106, Taiwan; yhchiu@tss.gov.tw (Y.-H.C.); ann850324@gmail.com (H.-P.W.); d08642003@ntu.edu.tw (W.-L.W.); kim15182@hotmail.com (Q.-W.S.); thanhha266@gmail.com (T.H.P.); 2 Seed Improvement and Propagation Station, Council of Agriculture, Taichung 427, Taiwan; 3 Institute of Plant Biology, National Taiwan University, Taipei 106, Taiwan; yuling7881@gmail.com; 4 Department of Engineering Science and Ocean Engineering, National Taiwan University, Taipei 106, Taiwan; ckhuang@ntu.edu.tw; 5 Agricultural Biotechnology Research Center, Academia Sinica, Taipei 115, Taiwan; 6 Center of Biotechnology, National Taiwan University, Taipei 106, Taiwan

**Keywords:** ABA signaling, calcium signaling, HTP-Seq, LTP-Seq, P1/HC-Pro^Tu^, stress response

## Abstract

The P1/HC-Pro viral suppressor of potyvirus suppresses posttranscriptional gene silencing (PTGS). The fusion protein of P1/HC-Pro can be cleaved into P1 and HC-Pro through the P1 self-cleavage activity, and P1 is necessary and sufficient to enhance PTGS suppression of HC-Pro. To address the modulation of gene regulatory relationships induced by turnip mosaic virus (TuMV) P1/HC-Pro (P1/HC-Pro^Tu^), a comparative transcriptome analysis of three types of transgenic plants (*P1^Tu^*, *HC-Pro^Tu^*, and *P1/HC-Pro^Tu^*) were conducted using both high-throughput (HTP) and low-throughput (LTP) RNA-Seq strategies. The results showed that P1/HC-Pro^Tu^ disturbed the endogenous abscisic acid (ABA) accumulation and genes in the signaling pathway. Additionally, the integrated responses of stress-related genes, in particular to drought stress, cold stress, senescence, and stomatal dynamics, altered the expressions by the ABA/calcium signaling. Crosstalk among the ABA, jasmonic acid, and salicylic acid pathways might simultaneously modulate the stress responses triggered by P1/HC-Pro^Tu^. Furthermore, the LTP network analysis revealed crucial genes in common with those identified by the HTP network in this study, demonstrating the effectiveness of the miniaturization of the HTP profile. Overall, our findings indicate that P1/HC-Pro^Tu^-mediated suppression in RNA silencing altered the ABA/calcium signaling and a wide range of stress responses.

## 1. Introduction

P1/HC-Pro is the first identified viral suppressor of potyvirus and can trigger the suppression of RNA silencing in the microRNA (miRNA) and short-interfering RNA (siRNA) regulatory pathways [1,2,3]. Our previous studies indicate that the FRNK motif of HC-Pro plays an essential role in the suppression of the miRNA pathway but still suppresses 40% of the siRNA pathway [2]. Moreover, Hu et al. (2020) demonstrated that various potyviral species of P1/HC-Pro, i.e., turnip mosaic virus (TuMV), zucchini yellow mosaic virus (ZYMV), and tobacco etch virus (TEV), have the same function in RNA silencing suppression [1]. However, the P1/HC-Pro of TuMV (P1/HC-Pro^Tu^) triggers ARGONAUTE1 (AGO1) degradation, whereas those of ZYMV (P1/HC-Pro^Zy^) and TEV (P1/HC-Pro^Te^) do not cause AGO1 degradation, which suggests that viral P1/HC-Pros exhibit functional diversity. Moreover, Sanobar et al. (2021) demonstrated that HC-Pro^Tu^ inhibits HEN1 activity in miRNA 3′-end 2′-*O*-methylation in vitro and in vivo through the binding activity of HC-Pro^Tu^ FRNK motif with HEN1 [4].

To understand P1/HC-Pro-mediated RNA silencing suppression further, a transcriptomic analysis based on transgenic Arabidopsis expressing the *P1/HC-Pro^Tu^* gene (*P1/HC-Pro^Tu^* plant) was previously performed via high-throughput (HTP) RNA sequencing (RNA-Seq) [1]. The transcriptomic profiles were subsequently analyzed through a comparative network using the ContigViews system. The network highlighted several critical gene silencing components, including *AGO1*, *AGO2*, and *AGO3*, as well as several miRNA targets, calcium signaling components, hormone signaling components, and defense response-related genes [1]. Hu et al. (2020) demonstrated that ethylene signaling genes in the *P1/HC-Pro^Tu^* plants are significantly highlighted in the gene-to-gene network and that endogenous ethylene is also highly accumulated in the *P1/HC-Pro^Tu^* plants [1]. Moreover, Pasin et al. (2020) showed that the P1 (P1^Pp^) of plum pox virus (PPV) triggers endogenous abscisic acid (ABA) accumulation in PPV-infected plants [5].

HTP RNA-Seq provides deep bioinformation; however, the abundant information obtained by RNA-Seq increases the analysis threshold for data mining and the difficulties in excluding the false-positive results generated with the low abundance gene profile. HTP RNA-Seq also has a higher cost for the deep sequencing. For example, the cost and sample determination for HTP RNA-Seq might limit the experimental design of a preliminary transcriptome study. Here, we propose the use of low-throughput (LTP) RNA-Seq in a preliminary study. The LTP RNA-Seq profiles were generated from *P1/HC-Pro^Tu^*-related transgenic plants and compared with the related P1/HC-Pro^Tu^-related profiles obtained previously via HTP RNA-Seq by Hu et al. [1].

In this study, we conducted the P1/HC-Pro^Tu^-related transcriptomic profiling using different logic analysis approaches to investigate the suppression mechanism further. We also performed LTP RNA-Seq of these P1/HC-Pro^Tu^-related materials and compared the networks obtained from the LTP datasets and previously published HTP profiles. The results indicate that LTP RNA-Seq has the potential to decrease the sequencing budgets and exclude genes with low expressions, which might yield a false-positive, and therefore, this approach could help researchers rapidly identify important pathways for further study.

## 2. Materials and Methods

### 2.1. Plant Materials and Transgenic Plants

*Arabidopsis thaliana* ecotype Col-0, three *P1/HC-Pro^Tu^*-related transgenic plants (*P1^Tu^*, *HC-Pro^T^^u^*, and *P1/HC*-*Pro^Tu^*), and *ago1-27* mutant were used in this study [1,6]. The *Arabidopsis* seeds were surface-sterilized, chilled at 4 °C for 2 days, and then sown on Murashige and Skoog (MS) medium with/without suitable antibiotics. All the plants were grown at 24 °C in a growth room with 16 h of light/8 h of dark.

### 2.2. cDNA Library Construction and RNA Sequencing

Ten-day-old and 14-day-old seedlings of the wild-type Col-0, *P1^Tu^*, *HC-Pro^Tu^*, and *P1/HC-Pro^Tu^* plants were used for the collecting samples for HTP and LTP whole-transcriptome deep sequencing, respectively. Three biological replicates of all the wild-type Col-0, *P1^Tu^*, *HC-Pro^Tu^*, and *P1/HC-Pro^Tu^* samples were included in this study, and each biological replicate consisted of 25–30 seedlings. Total RNA was extracted from the seedlings using a silica-gel membrane system (Viogene, New Taipei City, Taiwan). The mRNAs for LTP sequencing were isolated using the poly(A) mRNA magnetic isolation module (New England Biolabs, San Diego, CA, USA). All RNA sequencing libraries were constructed by using the cDNA library kit (Invitrogen Thermo Fisher Scientific, Waltham, MA, USA) according to the manufacturer’s instructions. Twelve cDNA libraries were constructed with three biological replicates of each sample. For HTP sequencing, the sequencing was accomplished by paired-end (2 × 125) strand-specific HiSeq sequencing (Illumina, San Diego, CA, USA), whereas for LTP, the sequencing was accomplished through paired-end (2 × 75) strand-specific MiSeq sequencing (Illumina) by next-generation sequencing (NGS) at the High Throughput Genomics Core of Academia Sinica. The clean reads were trimmed to filter out the adapters and the low-quality reads by using CLC Genomic Workbench (Qiagen, Hilden, Germany). The sequences of the coding regions in the whole Arabidopsis transcriptome were then mapped, and the reads were counted by using Bowtie 2 version 2.2.5 [7] and eXpress version 1.1.5 [8]. The raw RNA-Seq data of the HTP and LTP profiles are available at the National Center for Biotechnology Information Short Reads Archive (NCBI SRA) accession number SRR16916514-SRR16916533 and SRR16916443-SRR16916454 at the following URL: https://www.ncbi.nlm.nih.gov/Traces/study/?acc=PRJNA779609 and https://www.ncbi.nlm.nih.gov/Traces/study/?acc=PRJNA779601 respectively (accessed on 11 November 2021).

### 2.3. Differential Gene Expression Analysis and Functional Annotation

The transcriptome was analyzed using the ContigViews system (www.contigviews.bioagri.ntu.edu.tw, accessed on 11 November 2021) of the NGS core of National Taiwan University. The transcript abundances were based on read counts normalized to FPKM (fragments per kilobase per million). For the HTP expressional network analysis, the differentially expressed genes (DEGs) between the comparative Col-0 and *P1/HC-Pro^Tu^*-related datasets were identified based on an 80% passing rate, and genes with twofold log_10_ FPKM values less than 1.14 were filtered out. Due to the difference in the sequencing coverages achieved with HTP and LTP, different parameters were used in the ContigViews analysis. The LTP datasets were analyzed using appropriately adjusted parameter settings for the correlation thresholds during the construction of the expressional correlational networks. At least 10 samples from the Col-0, *P1^Tu^*, *HC-Pro^Tu^*, and *P1/HC-Pro^Tu^* profiles were selected to calculate the Pearson correlation based on a 0.975 threshold for a positive correlation and a 0.925 threshold for a negative correlation. For the LTP expressional network analysis, DEGs were identified using an 80% passing rate and a fold-change of 2, and the LTP networks were then constructed using thresholds of 0.95 and 0.90 for positive and negative correlations, respectively.

### 2.4. Quantification of Endogenous ABA and ABA Sensitivity Assay

Ten-day-old Arabidopsis seedlings (20–25 seedlings, approximately 60 mg for each extraction) were freshly collected and ground into fine powder with a tissue grinder pestle in a tube with liquid nitrogen. Fifty microliters of working solution (methanol) containing 0.5 ng of d_6_-ABA were added as a standard to each tube. After the addition of 500 µL of extraction solvent (2-propanol/H_2_O/concentrated HCl, 2:1:0.002), the tube was shaken at 100 r.p.m. and 4 °C for 30 min. Subsequently, 1 mL of dichloromethane was added to each tube, and the tube was shaken for another 30 min and centrifuged at 13,000× *g* and 4 °C for 5 min. Nine hundred microliters of the lower phase were transferred to a new tube, and the solvent was concentrated using a rotary evaporator (EYELA CVE-3110). An ultra-performance LC/ESI-qMS/MS analysis was conducted by the Metabolomic Core Facility at the Agricultural Biotechnology Research Center, Academia Sinica, Taiwan. A HALO C18 (Advanced Materials Technology, Inc., Wilmington, DE, USA) column (inner diameter, 2.1 mm; column length, 75 mm; particle size, 2.7 μm) was used, and gradient elution was performed with water and 0.05% glacial acetic acid (solvent A) and acetonitrile with 0.05% glacial acetic acid (solvent B) at a constant flow rate of 0.6 mL min^−1^. The following gradient profile was applied: t (min), % A): (0, 99), (2.20, 0), (2.40, 0), (2.60, 99), (3, 99). The MS and MS/MS experiments were performed with an API 3000 triple quadrupole mass spectrometer (PE Sciex, Concord, Ont., Canada) with the following parameters: temperature of 400 °C, nebulizer gas (N_2_) 10 (arbitrary units), curtain gas (N_2_) 12 (arbitrary units), collision gas (N_2_) 4 (arbitrary units), and the capillary voltage of −3.5 kV. The mass spectrometer was operated in multiple reaction mode (MRM). To germinate seeds for the ABA sensitivity assay, Arabidopsis seeds were sterilized and spread on the MS medium with or without 0.11 µM ABA, and the growth conditions were observed at 7 days after germination.

### 2.5. Real-Time Quantitative PCR

qRT-PCR was performed to validate the expression patterns of selected DEGs in the HTP network. Total RNA was extracted from each biological replicate of the Col-0, *P1/HC-Pro^Tu^*, and *P1/HC-Pro^Z^* plants (each replicate consisted of 25–30 seedlings) using a plant total RNA extraction miniprep system (Viogene-Biotek Corporation, New Taipei City, Taiwan). The obtained RNA was treated with a TURBO DNA-free Kit (Ambion Thermo Fisher Scientific, Waltham, MA, USA) and then subjected to phenol/chloroform extraction and alcohol precipitation to remove contaminating genomic DNA. First-strand cDNA was synthesized using MMLV reverse transcriptase (Invitrogen, Carlsbad, CA, USA). Gene-specific primers for the DEGs were designed using Primer3Plus [9]. The primer sets of OZF1_qPCR_F1 (5′-CGGATTCGTAAACCGGAGTGTCTG-3′) and OZF1_qPCR_R1 (5′-GAGGAATCTCCCTCGAATCATCGATTATG-3′) for *OZF1*, MYB44_qPCR_F1 (5′-GGAGTTGGGAGAATCGAGTAGACAAAGTG-3′) and MYB44_qPCR_R1 (5′-CGTCACTACGTCCCCAGCTCTC-3′) for *MYB44*, MYB96_qPCR_F1 (5′-GCTCTACAACACTCTTTTCCCCTTTTGG-3′) and MYB96_qPCR_R1 (5′-GCATAACCATATGAGCCACAAAGTGAAAC-3′) for *MYB96*, ABF4_qPCR_F1 (5′-TGGTGCAAATGAGGCCATGATTGG-3′) and ABF4_qPCR_R1 (5′-GGCAAAACAAATCATGCAGTGTACCTG-3′) for *ABF4*, IQM4_qPCR_F1 (5′-GCCTTGTCAACTTAACTCACCAAGAAGTG-3′) and IQM4_qPCR_R1 (5′-CCTTGGGCATTTCACCTAAACCAGAAG-3′) for *IQM4*, CaLB_qPCR_F1 (5′-TCCTTGGTTTTGTGTGTTCATCATCCTC-3′) and CaLB_qPCR_R1 (5′-GCGATGATTATACGCCGATAAGTTCCG-3′) for *CaLB*, CPK28_qPCR_F1 (5′-CGCAGCAAAACAAAGAGAGAAAGTGG-3′) and CPK28_qPCR_R1 (5′-ATTCAGGGAATGCCACGTGTCCTC-3′) for *CPK28* and P_Actin2 (5′-CCTCAATCTCATCTTCTTCCGCTC-3′) and M_Actin2 (5′-AGCATCATCTCCTGCAAATCCAGC-3′) for *ACT2* were used for expressional detection. The qRT-PCR assays were performed using the Light Cycler 480 System (Roche) with the KAPA SYBR FAST qPCR Master Mix (2×) Kit (Sigma-Aldrich, St. Louis, MO, USA). Three biological replicates and three technical replicates were included in the assays. The expression levels of *ACT2* were used as the internal control, and normalized mRNA expression levels were calculated using the formula 2^−ΔCt^.

### 2.6. Expression-Based Heatmaps and Principal Component Analysis (PCA)

The identified DEGs were functionally annotated based on their sequence similarities with known protein annotations in the public TAIR database (www.arabidopsis.org, accessed on 11 November 2021). Heatmaps of DEG expressions were generated with the ClustVis web tool [10]. PCA was performed using the PCA method of the decomposition module in the scikit-learn package of Python. The principal component (PC) scores were plotted with the Matplotlib package. The PCA analysis was performed using the FPKM values for all the transcripts obtained from individual replicates. Thus, the PCA-based comparison of gene expression was performed using 24 libraries of the 4 Col-o, *P1^Tu^*, *HC-Pro^Tu^*, and *P1/HC-Pro^Tu^* samples from the HTP and LTP datasets.

## 3. Results

### 3.1. P1/HC-Pro^Tu^ Suppressor Triggers Plant Defense Responses

**Figure 1:**
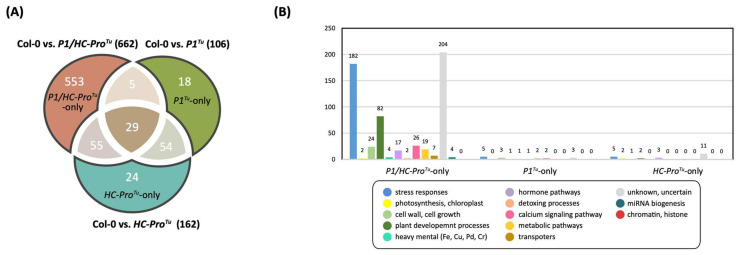
Network genes among the three comparison datasets obtained from the HTP RNA-seq profiling: Col-0 vs. *P1/HC-Pro^Tu^*, Col-0 vs. *P1^Tu^*, and Col-0 vs. *HC-Pro^Tu^* comparative sets. (**A**) Venn diagram showing the distributions of shared and unique network genes. (**B**) Functional classification of unique genes in the *P1^Tu^*-only, *HC-Pro^Tu^*-only, and *P1/HC-Pro^Tu^*-only sections.

The comparative analyses of the HTP RNA-Seq profiles of Col-0 vs. *P1/HC-Pro^Tu^*, Col-0 vs *P1^Tu^*, and Col-0 vs. *HC-Pro^Tu^* sets revealed 1601, 559, and 777 DEGs, respectively (Table 1). These DEGs were then used for a network analysis using the ContigViews system, which revealed 662, 106, and 162 genes in the networks, respectively (Table 1). A Venn diagram showed that 29 of the network genes were found in all three comparative sets (Figure 1A). The *P1/HC-Pro^Tu^*-only section contained a markedly higher number of network genes (553), whereas the *P1^Tu^*-only and *HC-Pro^Tu^*-only sections contained only 18 and 24 genes, respectively (Figure 1A).

To elucidate the functions of the unique genes in the *P1/HC-Pro^Tu^*-only, *P1^Tu^*-only, and *HC-Pro^Tu^*-only sections further, we performed gene annotation and functional classification. Unique genes with functions related to stress-responses, cell growth, and plant development were predominantly enriched in the *P1/HC-Pro^Tu^*-only section in addition to unknown or unclassified functional genes (Figure 1B). Similar gene functional classification results were found for the *P1^Tu^*-only and *HC-Pro^Tu^*-only sections, although only 18 and 24 genes were identified in these two sections, respectively (Figure 1A). A high abundance of network genes in the *P1/HC-Pro^Tu^*-only section were classified as being involved in ABA phytohormone and calcium signaling pathways (Figure 1A).

### 3.2. P1/HC-Pro^Tu^ Alters ABA-Induced Immune Responses

**Figure 2:**
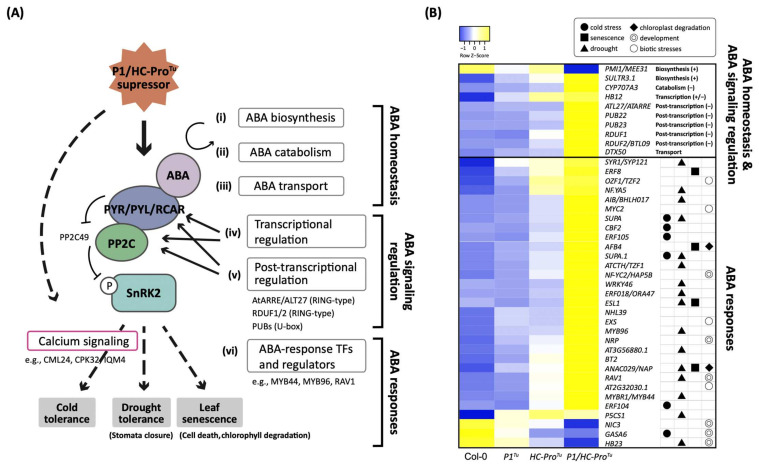
The P1/HC-Pro^Tu^ altered ABA-mediated immune responses. (**A**) *P1/HC-Pro^Tu^*-only genes in the *P1/HC-Pro^Tu^*-only section of the HTP datasets that were functionally annotated to categories and subcategories of genes involved in (i) ABA biosynthesis, (ii) catabolism, (iii) transport, (iv) signaling regulation, (v) posttranscriptional regulation, (vi) calcium signaling, and (vii) ABA responses. (**B**) Heatmap showing the expression patterns of the genes in the *P1/HC-Pro^Tu^*-only section that are involved in the ABA signaling pathway. ‘+’ and ‘−’ represent genes that positively or negatively regulate the ABA signaling pathway. The symbols in the right panel indicate the functions of genes in various biotic and abiotic stress responses and developmental processes.

ABA plays pivotal roles in seed germination and stress tolerance [11]. Several studies demonstrate that abiotic stresses increase ABA and Ca^2+^ concentrations in the cytosol and induce signaling to modulate common target proteins (Figure 2A) [12]. We found 41 genes belonging to the ABA signaling pathway that could be further classified into the ABA homeostasis, ABA signaling regulation, and ABA response categories (Figure 2A and Table 2). Among these genes, *PMI1/MEE31* (AT3G02570) and *SULTR3;1* (AT3G51895) that belong to the ABA positive biosynthesis regulator were respectively repressed and induced in the *P1/HC-Pro^Tu^* plants (Figure 2A(panel i),B). The expression of both *CYP707A3* (AT5G45340), involved in ABA catabolism, and *DTX50* (AT5G52050), involved in ABA transport, was induced in the *P1/HC-Pro^Tu^* plants (Figure 2A(panel ii and iii),B). As for the ABA signaling regulation, *RCAR/PYLs*, *PP2C*, and *SRNK2*, the core components of ABA signaling transduction, were not differentially expressed in our study, even though several negative regulators (e.g., *ATL27/ATARRE* (AT5G66070), *PUB22* (AT3G52450), *RDUF1* (AT3G46620), *RDUF2* (AT5G59550), and *HB12* (AT3G61890)), which inhibit RCAR/PYL and PP2C expressions either transcriptionally or posttranscriptionally, had an induced expression pattern in the *P1/HC-Pro^Tu^* plants (Figure 2A(panel iv and v),B).

ABA response genes accounted for the highest proportion of the genes involved in the ABA signaling pathway found in the *P1/HC-Pro^Tu^*-only section and could be divided into the biotic stress response, development, drought stress response, cold stress response, and senescence subcategories (Figure 2A(panel vi and vii) and Table 2). Almost all of these ABA response genes were expressed at a higher level in the *P1/HC-Pro^Tu^* plants than in Col-0, *P1^Tu^*, and *HC-Pro^Tu^* plants (Figure 2B). Induced expressions of these ABA response genes might change plant resistance to drought stress, cold stress, and leaf senescence in response to P1/HC-Pro^Tu^. These results revealed that the regulation of the ABA signaling pathway was disrupted, and its responses might be severely interfered with overexpressing *P1/HC-Pro^Tu^*.

### 3.3. Quantification of Endogenous ABA and ABA Sensitivity Assay

**Figure 3:**
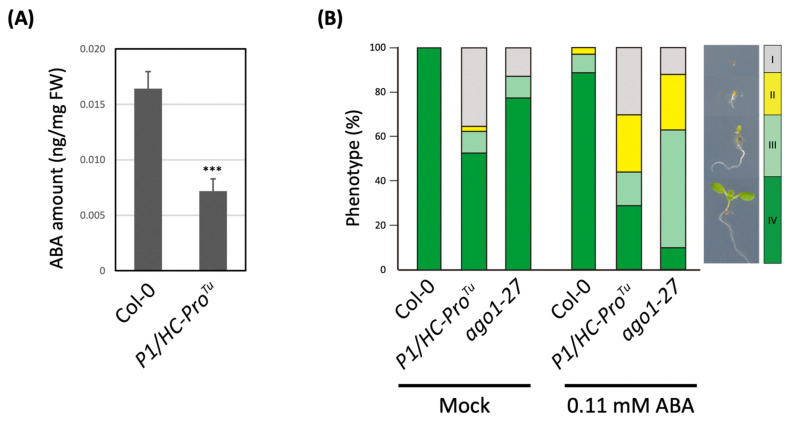
Endogenous ABA detection and ABA sensitivity assay. (**A**) Determination of the endogenous ABA amounts. The mean values ± *SD* were obtained from three biological repeats. Comparisons between two groups were performed using a Student’s *t* test. *** *p* < 0.001. (**B**) ABA sensitivity assay. The germination phenotypes were classified into four phases: I, rupture of the seed coat; II, radicle protrusion; III, fully-opened cotyledons; and IV, true leaf development.

To examine ABA accumulation in the *P1/HC-Pro^Tu^* plants, the 10-day-old seedlings were extracted and measured by MS/MS analysis. A significantly lower ABA amount was detected in the *P1/HC-Pro^Tu^* seedlings than in the Col-0 (Figure 3A). To test the effects of ABA on the *P1/HC-Pro^Tu^* seedlings further, an ABA sensitivity assay was carried out to observe the phenotypical changes with seed germination. P1/HC-Pro^Tu^ plays a major role in PTGS suppression by triggering AGO1 degradation [1]. Therefore, *ago1-27* mutant was also used for the ABA sensitivity assay. Without ABA treatment, the seeds of Col-0 were germinated and developed into true-leaf seedlings at phase IV, while seeds of the *P1/HC-Pro^Tu^* plants and *ago1-27* mutant were more late-germinated or delayed-growth at phase I, II, and III (Figure 3B), suggesting that the germination rate and post-germination growth were greatly delayed in the *P1/HC-Pro^Tu^* plants and *ago1-27* mutant. With exogenous ABA treatment, the delayed-germination phenotype became more severe in the *P1/HC-Pro^Tu^* plants and *ago1-27* mutant than in the Col-0 (Figure 3B). Specifically, more than half of the *P1/HC-Pro^Tu^* seeds remained at phases I and II, which indicated that the *P1/HC-Pro^Tu^* plants exhibited a higher sensitivity to ABA during seed germination.

### 3.4. P1/HC-Pro^Tu^ Triggers Immune Responses in a Calcium-Dependent Manner

**Figure 4:**
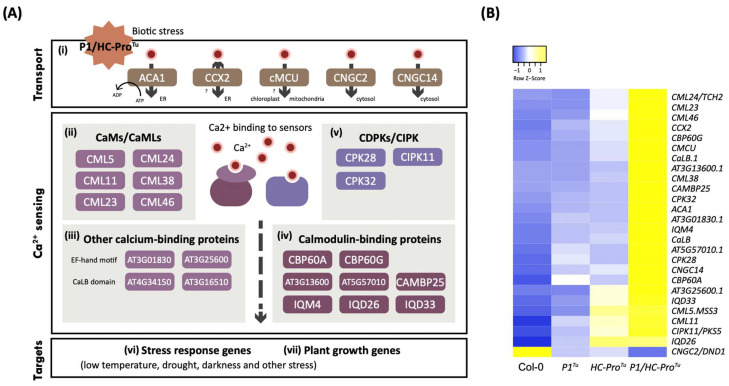
Calcium signaling pathway in response to P1/HC-Pro*^Tu^*. (**A**) Calcium signaling pathway in the *P1/HC-Pro^Tu^*-only section of the HTP profiles in the Col-0 vs. *P1/HC-Pro^Tu^*, Col-0 vs. *P1^Tu^*, and Col-0 vs. *HC-Pro^Tu^* comparison sets obtained from the HTP profiles. (**B**) Heatmap showing the expression patterns of the *P1/HC-Pro^Tu^*-only genes involved in the calcium signaling pathway.

We found that the genes in the *P1/HC-Pro^Tu^*-only section encode various Ca^2+^ transporters, including Ca^2+^ channels [*CMCU* (AT5G66650), *CNGC2* (AT5G15410), and *CNGC14* (AT2G24610)], Ca^2+^ co-transporter [*CCX2* (AT5G17850)], and Ca^2+^ pump [*ACA1* (AT1G27770)] (Figure 4A(panel i) and Table 3). We also identified several Ca^2+^ sensors, including Ca^2+^/calmodulin (CaM), CaM-like (CaML) (Figure 4A(panel ii)), calcium binding proteins (Figure 4A(panel iii)), calmodulin-binding proteins (Figure 4A(panel iv)), and CDPKs/CIPKs (Figure 4A(panel v) and Table 3). The members of the various families of Ca^2+^ sensors identified in the *P1/HC-Pro^Tu^*-only section were expected to contribute to the conversion of Ca^2+^ signals into either cellular stress responses or developmental processes (Figure 4A(panel vi and vii)). For instance, the expressions of genes encoded calmodulin-binding proteins, including *CAMBP25* (AT2G41010) and *IQM4* (AT2G26190), whose gene expressions are induced by drought and salt/osmotic stress. *CPK32* (AT3G57530) and *CPK28* (AT5G66210) encode calcium-dependent protein kinases that regulate plant growth in addition to resetting pathogen-associated molecular pattern (PAMP)-induced defense signaling. The majority of genes involved in the Ca^2+^ signaling pathway were expressed at higher levels in the *P1/HC-Pro^Tu^* plants than in the Col-0, *P1^Tu^*, and *HC-Pro^Tu^* plants (Figure 4B) except for *CNGC2* (AT5G15410), which suggests that the induction of the calcium signaling pathway depends on ectopic-expressing P1/HC-Pro^Tu^. In summary, the results indicate that P1/HC-Pro^Tu^ might trigger various stress responses and developmental processes through the calcium signaling pathway.

### 3.5. Validation of DEGs in the ABA and Ca^2+^ Pathways

**Figure 5:**
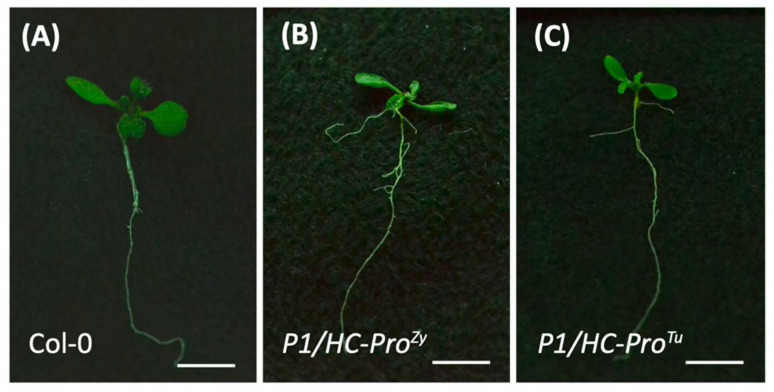
Phenotypes of the *P1/HC-Pro^Tu^* and *P1/HC-Pro^Zy^* plants. Ten-day-old seedlings of (**A**) Col-0, (**B**) *P1/HC-Pro^Zy^*, and (**C**) *P1/HC-Pro^Tu^* transgenic plants. Scale bars = 0.5 cm.

**Figure 6:**
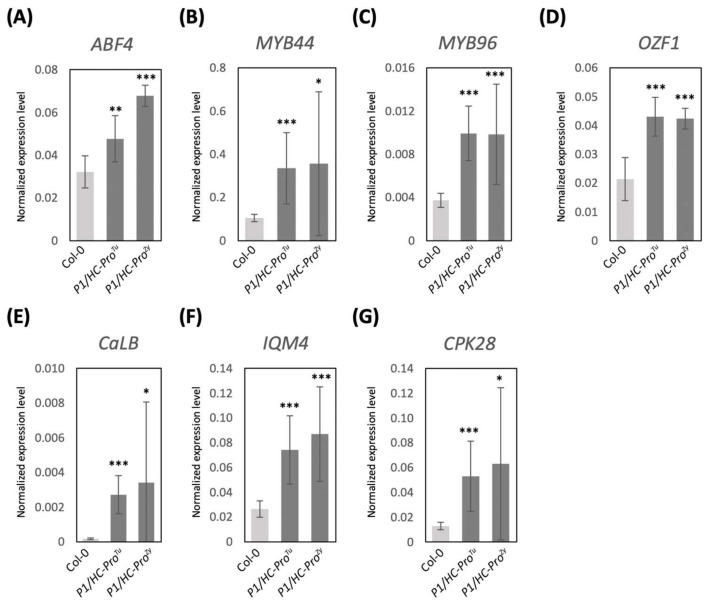
The qRT-PCR-based validation of the gene expressions in the *P1/HC-Pro*-related plants obtained from the HTP profiles. (**A**–**D**) DEGs in the ABA signaling pathway. (**E**–**G**) DEGs in the Ca^2+^ signaling pathway. The mean values ± *SD* were from three biological repeats. Comparisons between two groups were performed with Student’s *t* test. * *p* < 0.05, ** *p* < 0.01, *** *p* < 0.001.

The expression patterns of several candidate genes in the ABA and Ca^2+^ signaling pathways were validated by qPCR. In addition to the Col-0 and *P1/HC-Pro^Tu^* plants, *P1/HC-Pro^Zy^* plants were also included in gene expressional validation. Compared with the ovate leaves of the Col-0 plants, the serrated-leaf phenotype was observed in the *P1/HC-Pro^Tu^* and *P1/HC-Pro^Zy^* seedlings, which implies common PTGS suppression effects induced by the heterogeneous P1/HC-Pros. (Figure 5). Four ABA response genes (*ABF4*, *MYB44*, *MYB96*, and *OZF1*) exhibited similar expression patterns with higher transcripts in the *P1/HC-Pro^Tu^* plants, consistent with the HTP RNA-Seq profiles. Moreover, the expression levels of the selected genes were also highly induced in the *P1/HC-Pro^Zy^* plants (Figure 6A–D). Additionally, *CaLB*, *IQM4*, and *CPK28* involved in the Ca^2+^ signaling pathway showed similar qRT-PCR results to those of the ABA response genes (Figure 6E–G). The qRT-PCR results indicate that the overall expressions of genes in the ABA and Ca^2+^ signaling pathways were consistent in the *P1/HC-Pro^Tu^* and *P1/HC-Pro^Zy^* plants.

### 3.6. P1/HC-Pro^Tu^ Triggers Drought Response and Stomatal Closure

**Figure 7:**
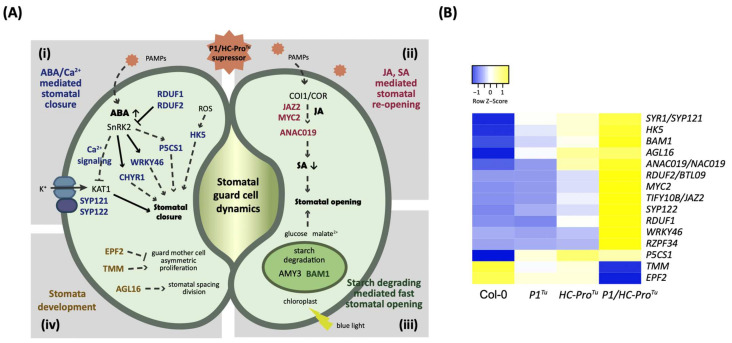
Regulatory mechanism controlling stomatal dynamics in response to P1/HC-Pro^Tu^. (**A**) Genes involved in the regulation of stomatal guard cell dynamics and development found in the *P1/HC-Pro^Tu^*-only section. The genes involved in stomatal closure, stomatal opening, starch degradation, and stomatal development are labeled in blue, red, green, and brown, respectively. (**B**) Heatmap showing the expression patterns of the *P1/HC-Pro^Tu^*-only genes involved in the stomatal closure, opening, and development.

Our data mining of the *P1/HC-Pro^Tu^*-only section revealed 66 drought stress-related genes, e.g., *MYB96* (AT5G62470), *MYB44* (AT5G67300), *NF-YA5* (AT1G54160), *ANAC029* (AT1G69490), and *TZF1* (AT2G25900), that were highly enriched in terms annotated to drought stress responses (Table 4). Among them, 21 and 4 genes were found to be involved in either the ABA or Ca^2+^ signaling pathway, respectively (Table 4). Notably, four regulatory modules composed of 15 drought response genes that function in controlling stomatal guard cell dynamics were identified (Figure 7A and Table 4). Genes in these regulatory modules could induce ABA/Ca^2+^-mediated stomatal closure, salicylic acid (SA)- or jasmonic acid (JA)-mediated stomatal opening, starch degradation-mediated rapid stomatal reopening, and guard cell division to influence stomatal development (Figure 7A). The mechanisms through which these genes are controlled and the involvement of phytohormones and environmental stimuli that are involved in the regulation of stomatal dynamics are explained below.

In the ABA/Ca^2+^-mediated stomatal closure modulation (Figure 7A(panel i)), genes such as *WRKY46* (AT2G46400) and *RZPF34/CHYR1* (AT5G22920) function in the response to ABA and to water deficit stress (Figure 5A). However, several genes, such as *JAZ2*, *MYC2*, and *ANAC019* (AT1G74950, AT1G32640, and AT1G52890), modulate stomatal reopening after microbe-associated molecular pattern (MAMP)-mediated stomatal closure and activate the JA pathway upon bacterial infection (Figure 7A(panel ii)). Similarly, stomatal reopening is modulated by either the SA-mediated pathogen infection signaling pathway (Figure 7A(panel ii)) or light-induced starch degradation by the glucan hydrolase β-AMYLASE1, which is encoded by *BAM1* (AT3G23920), to promote rapid stomatal opening (Figure 7A(panel iii)). All the DEGs in the three modulations showed greater induced expression levels in the *P1/HC-Pro^Tu^* plants than in the Col-0, *P1^Tu^*, and *HC-Pro^Tu^* plants (Figure 7B).

Moreover, the stress responses induced by P1/HC-Pro^Tu^ could affect the regulatory mechanisms of stomatal development and lead to stable long-term adaptations to stress (Figure 7A(panel iv)). Induced expression of the negative regulator *EPF2* (AT1G34245) and the positive regulator *TMM* (AT1G80080) might affect the asymmetric divisions of guard mother cells (Figure 7B). An accumulation of *AGL16* (AT3G57230) with silent mutations (*AGL16m*) in the miR824 recognition site reportedly promotes the development of higher-order stomatal complexes by increasing the number of additional divisions in meristemoid cells [13]. With the exceptions of *EPE2* and *TMM*, all of these genes were expressed at higher levels in the *P1/HC-Pro^Tu^* plants than in the Col-0, *P1^Tu^*, and *HC-Pro^Tu^* plants (Figure 7B). Overall, the results indicate that the integration of ABA, calcium, and other hormone signals could simultaneously trigger dynamic closure and opening mechanisms during drought stress or biotic stress in the *P1/HC-Pro^Tu^* plants.

### 3.7. P1/HC-Pro^Tu^ Stimulates Cold Response and Leaf Senescence

**Figure 8:**
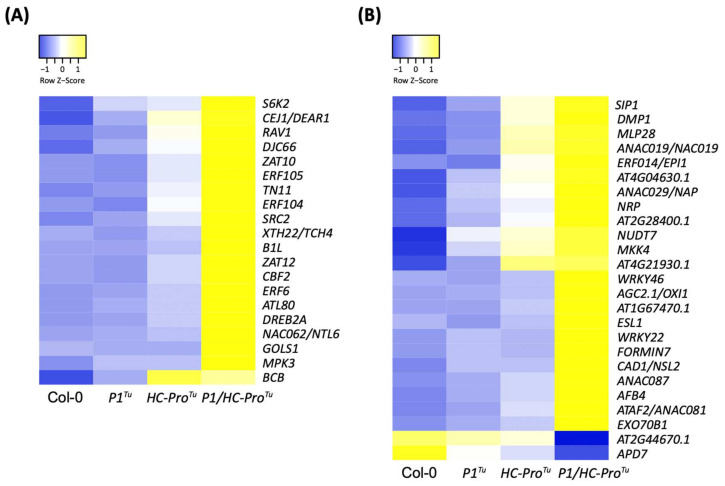
Heatmaps showing the expression patterns of (**A**) cold response- and (**B**) senescence-related genes in the *P1/HC-Pro^Tu^*-only section of the HTP datasets.

Twenty cold response genes were identified in the *P1/HC-Pro^Tu^*-only section obtained from of the HTP profiles (Table 5). All of these genes showed higher expression levels in the *P1/HC-Pro^Tu^* plants that were different from those found in the Col-0, *P1^Tu^*, and *HC-Pro^Tu^* plants (Figure 8A). Because a transient increase in cytosolic Ca^2+^ levels is detected within seconds of cold shock [14], several annotated cold-related genes in our dataset, including *CBF2* (AT4G25470), *CEJ1/DEAR1* (AT3G50260), *ZAT10* (AT1G27730), and *ZAT12* (AT5G59820), were found to be involved in the cold stress-induced Ca^2+^ signature (Table 5). Moreover, seven of the cold-response genes were involved in the drought stress response (Table 5), which indicates that the gain of *P1/HC-Pro^Tu^* function may suggest overlaps between the gene regulatory mechanisms underlying enhancement to cold and drought tolerance. However, there were still many cold-response genes that were independent of the ABA/Ca^2+^ signaling pathways, suggesting that *P1/HC-Pro^Tu^* could stimulate a cold response via other regulating mechanisms.

Although leaf senescence is a general developmental program, it can be induced by abiotic and biotic stresses through the transcriptional control of senescence-related transcription factors [15]. Twenty-five genes annotated as being involved in leaf senescence, cell death, and chlorophyll degradation were identified in the *P1/HC-Pro^Tu^*-only section (Table 6). Among these genes, the *WRKY22* (AT4G01250) and *ERF014* (AT1G44830) transcription factor-encoding genes function in mediating dark-induced senescence [16,17]. Chlorophyll degradation is one of the most visually apparent phenomena that occurs during leaf senescence [18,19]. The leaf degreening process appears to be promoted by genes involved in the ABA signaling pathway [*ABF4* (AT4G24390)] as well as in the JA [*ANAC019* (AT1G52890)] and GA [*ANAC029* (AT1G69490)] signaling pathways. Developmentally controlled programmed cell death occurs at the terminal stage of leaf senescence [18] The identified *NUDT7* (AT4G12720) and *CAD1* (AT1G29690) genes might play distinct roles in plant immunity related to the regulation of programmed cell death [20,21]. With the exceptions of *APD7* (AT5G02760) and *AT2G44670*, all of these genes were expressed at higher levels in the *P1/HC-Pro^Tu^* plants than in the Col-0, *P1^Tu^* and *HC-Pro^Tu^* plants (Figure 8B). Taken together, our results suggest that cold tolerance, chloroplast degradation, and leaf senescence are likely to serve as key responses in the *P1/HC-Pro^Tu^* plants.

### 3.8. Comparison of the HTP and LTP Profiles

We then compared the data mining efficiency of the LTP profile (~2 M reads) with that of the HTP profile (~ 20 M reads). A total of 21.94 to 24.66 M clean reads were generated from all the samples for the HTP profiles, and these exhibited an average mapping rate of 79.2% to the coding DNA sequence (CDS) (Table 7). Approximately one-tenth of the total sequencing depth was used to construct the LTP profiles; thus, the LTP profiles contained 1.89 to 2.96 M clean reads obtained from 1.97 to 3.10 M raw reads (Table 7). The average mapping rate of the LTP profiles was 78.5%, which was close to that found for the HTP profiles (Table 7). The equivalent mapping rates obtained for the HTP and LTP libraries indicate that the mapping ability of the RNA-Seq reads does not depend on the RNA-Seq depth.

**Figure 9:**
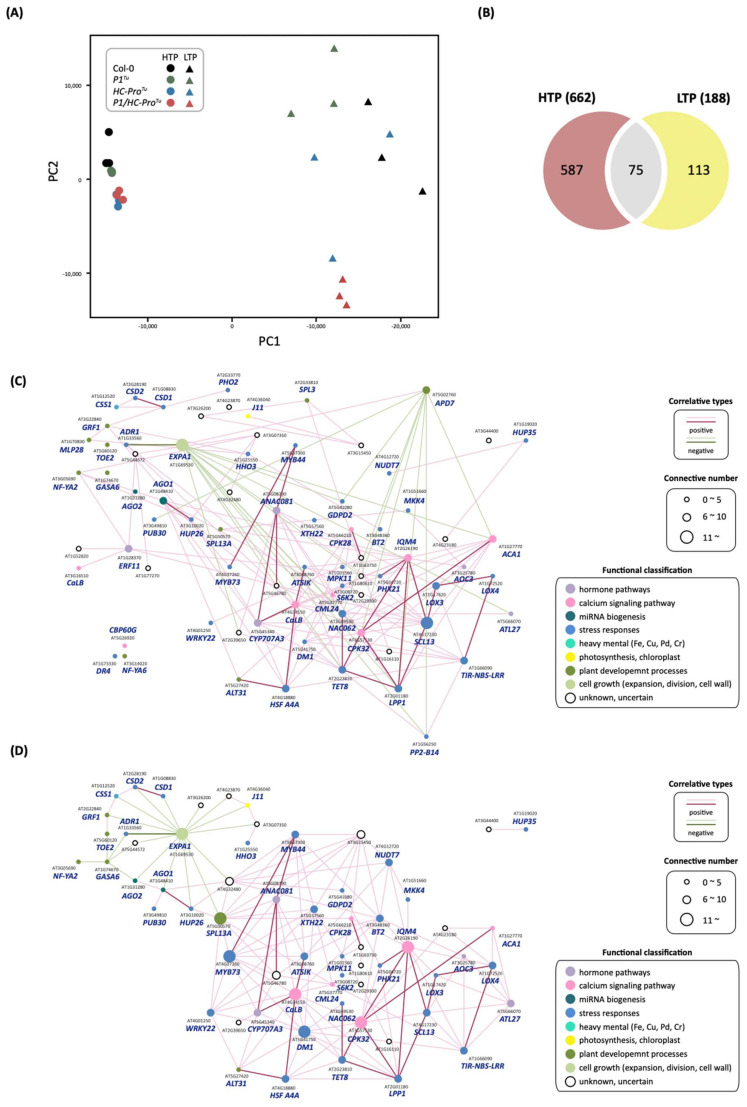
Comparison of gene expression and networks obtained with the HTP and LTP profiles. (**A**) PCA plot of gene expression profiles from the HTP and LTP datasets of Col-0, *P1^Tu^*, *HC-Pro^Tu^*, and *P1/HC-Pro^Tu^* samples. The top two principal components (*X*-axis, PC1; *Y*-axis, PC2) are shown. Each data point represents one biological sample. (**B**) Venn diagram showing the shared and unique genes in the Col-0 vs. *P1/HC-Pro^Tu^* comparison samples between the HTP and LTP datasets. Network-based comparison of the 75 shared genes in the Col-0 vs. *P1/HC-Pro^Tu^* datasets between (**C**) the HTP and (**D**) the LTP datasets. The red and green lines represent positive and negative correlations, respectively. The dark red and green lines indicate conserved connections between the HTP and LTP networks, respectively.

We then performed a PCA of the HTP and LTP profile data (Figure 9A). The top two PCs explained 75.7% of all differences among the three varieties, and PC1 accounted for 63.0%, which suggested that PC1 can distinguish between the HTP and LTP profiles (Figure 9A). We also noted that biological replicates of the HTP profiles were more consistent than those of the LTP profiles (Figure 9A). Furthermore, the PCA clustering of the HTP data corresponded to the morphological phenotypes: the Col-0 and *P1^Tu^* plants had identical normal developmental phenotypes, whereas the *HC-Pro^Tu^* and *P1/HC-Pro^Tu^* plants had a serrated leaf phenotype. In contrast, PC2 explained only 12.7% of the overall differences but was most likely to distinguish the *P1/HC-Pro^Tu^* samples from the other samples (Figure 9A). In addition, based on PC2, the clustering of the *P1/HC-Pro^Tu^* samples distinctly differed from that of the other samples, and this finding was obtained for both the HTP and LTP profiles.

**Figure 10:**
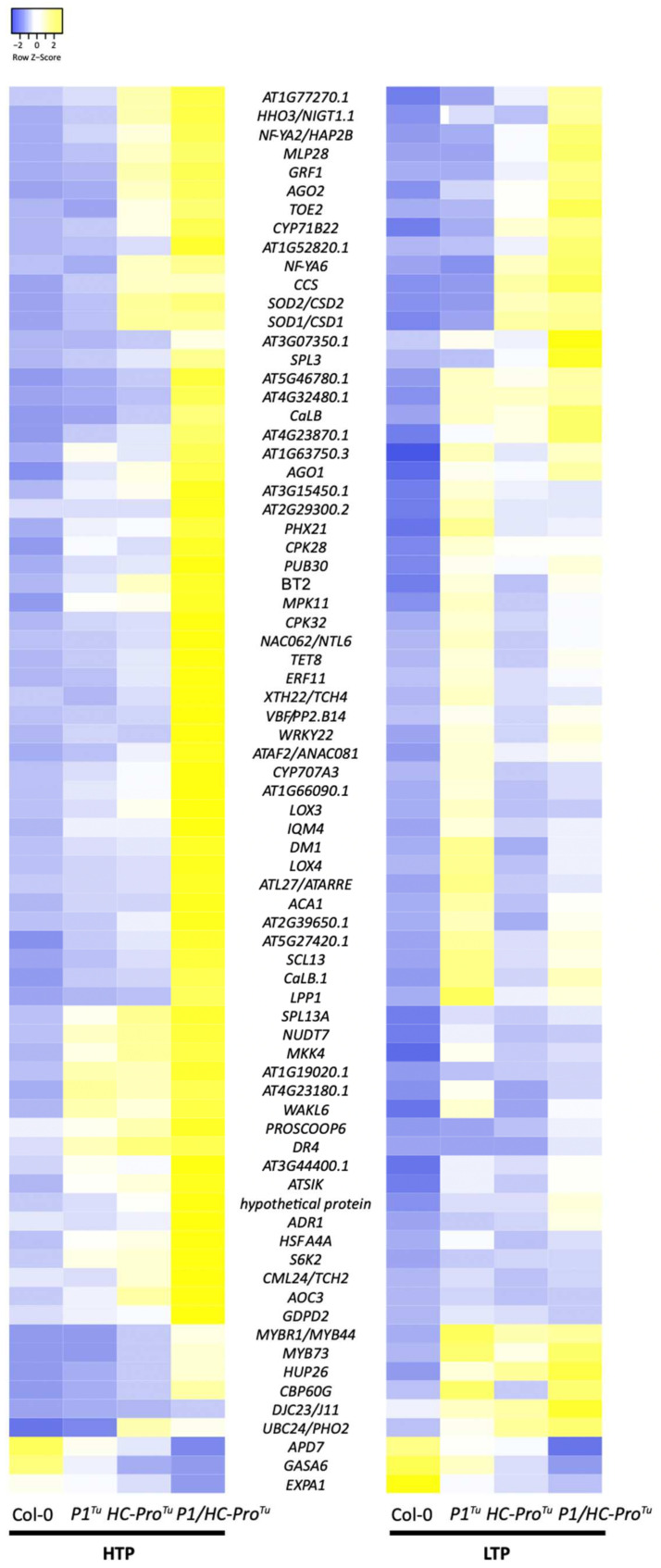
Heatmaps showing the expression patterns of the 75 overlapped genes in the Col-0 vs. *P1/HC-Pro^Tu^* comparison identified from both the HTP and LTP profiles.

We compared the Col-0 vs. *P1/HC-Pro^Tu^* plant samples, and the results revealed 75 common genes, which were shown in the intersection area of the networks obtained with the HTP and that obtained with the LTP profiles (Figure 9B and Table 8). These genes were characterized as being involved in ABA/Ca^2+^ signaling pathways, drought or cold stress responses, senescence, and gene silencing and RNA regulation (Table 8). We also found that the 75 common genes were located at identical positions in the HTP and LTP networks for comparison (Figure 9C,D). Moreover, the HTP and LTP profile-based networks of the 75 common genes revealed 132 and 159 gene-gene correlations for the HTP and LTP profiles, respectively (Figure 9C,D). However, we observed that connections associated with the positive and negative correlations were not 100% identical between the HTP and LTP profiles (Figure 9C,D). Twenty-six correlations (19.7%), including 25 positive connections and one negative connection, among the 30 common genes in the HTP network remained conserved in the LTP network. Furthermore, the heatmaps of the 75 common genes in the HTP and LTP profiles exhibited similar expression patterns, and the expressions of these genes were upregulated in the *P1/HC-Pro^Tu^* plants (Figure 10).

### 3.9. Functional Classification of the DEGs Identified from the LTP Profile

**Figure 11:**
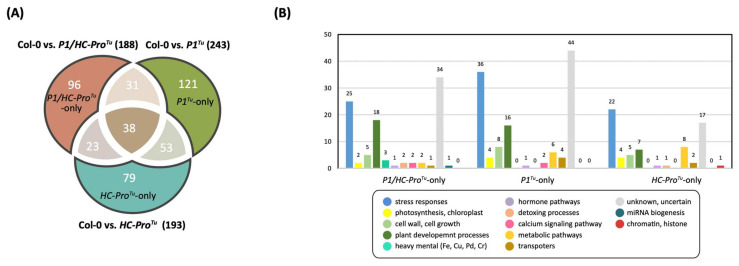
Network genes among the three comparison sets obtained from the LTP profiles: Col-0 vs. *P1/HC-Pro^Tu^*, Col-0 vs. *P1^Tu^*, and Col-0 vs. *HC-Pro^Tu^* comparison sets. (**A**) Venn diagram showing the distributions of shared and unique network genes. (**B**) Functional classification of unique genes in the *P1^Tu^*-only, *HC-Pro^Tu^*-only, and *P1/HC-Pro^Tu^*-only sections.

A total of 188, 234, and 193 network genes were identified in the Col-0 vs. *P1/HC-Pro^Tu^*, Col-0 vs. *P1^Tu^*, and Col-0 vs. *HC-Pro^Tu^* LTP comparison sets, respectively, whereas the corresponding HTP comparison sets contained 553, 18, and 24 network genes, respectively (Table 1). The LTP dataset revealed equivalent gene numbers among the three comparison sets, whereas the HTP dataset showed a higher abundance of network genes in the Col-0 vs. *P1/HC-Pro^Tu^* comparison. A Venn diagram was generated to determine the unique and shared genes among the Col-0 vs. *P1/HC-Pro^Tu^*, Col-0 vs. *P1^Tu^*, and Col-0 vs. *HC-Pro^Tu^* comparison sets. Sixty-nine shared network genes were identified from the three comparison sets of the LTP profiles (Figure 11A). Adequate gene numbers were also obtained in the *P1/HC-Pro^Tu^*-only (96 genes), *P1^Tu^*-only (121 genes), and *HC-Pro^Tu^*-only (79 genes) sections (Figure 11A). Moreover, functional characterization revealed that genes involved in stress responses, plant development processes, and the calcium signaling pathway were abundant in the *P1/HC-Pro^Tu^*-only section obtained with the LTP profiles, which are similar to the results obtained from the functional characterization of genes in the *P1/HC-Pro^Tu^*-only section based on the HTP profiles (Figure 1B and Figure 11B). Notably, the *P1^Tu^*-only and *HC-Pro^Tu^*-only sections obtained from the HTP and LTP profiles were not significantly identical (Figure 1B and Figure 11B).

## 4. Discussion

### 4.1. P1/HC-Pro^Tu^ Alters ABA and the Other Hormones Accumulations

Multiple plant hormones are reported to respond to P1/HC-Pros [1,5]. Endogenous ethylene is maintained at a higher level in the *P1/HC-Pro^Tu^* plants, and the comparative network of Col-0 vs. *P1/HC-Pro^Tu^* also highlighted critical genes in various hormone signalings (e.g., JA, ethylene, and ABA) [1]. Hu et al. (2020) also proposed that the serrated leaf phenotype of the *P1/HC-Pro^Tu^* plants might relate to the endogenous auxin accumulation [1]. These studies implied a comprehensive alternation among different hormone pathways that occurred in response to P1/HC-Pros. Consequently, the coordinated modulations or crosstalk of hormone responses could possibly be interfered by P1/HC-Pros and cause changes in growth and immunity responses.

In this study, the ABA signaling pathway was fundamentally changed in the *P1/HC-Pro^Tu^* plants. For example, *P1/HC-Pro^Tu^* triggered the ABA negative regulator up-regulation and interfered with ABA positive regulator expressions for the ABA homeostasis and signaling regulation, resulting in low abundant endogenous ABA in the *P1/HC-Pro^Tu^* plants. Surprisingly, the ABA response genes were mostly induced in the *P1/HC-Pro^Tu^* plants (Figure 2), implying that the PTGS suppression might alter these gene expressions. Indeed, the endogenous AGO1 was degraded in the *P1/HC-Pro^Tu^* plants [1], which also showed ABA-sensitivity in seed germination as *ago1-27* mutants, suggesting that AGO1 deficiency might disrupt ABA sensing and ABA responses. However, not P1/HC-Pros of all viral species have the same effect in ABA pathway regulation. Pasin et al. (2020) show that P1^Pp^ increases the amounts of ABA [5], which is conflicted with the ABA profile of P1/HC-Pro^Tu^. Our explanation is the sequence divergence of P1^Pp^ and P1^Tu^, which showed only 19.35% amino acid identity, resulting in the difference in endogenous ABA accumulation.

### 4.2. P1/HC-Pro^Tu^ Might Alter ABA and Calcium Signaling Crosstalk during Stomatal Closure and Drought Stress

Abiotic stress and biotic stress can initiate ABA signaling pathways that lead to many molecular and cellular responses [22,23]. Among the ABA-induced stress response genes, those controlling stomatal closure and opening are important during drought conditions [24], and stomatal immunity plays an important role in the restriction of pathogen entry [24]. Thus, stomatal movement serves as a platform for crosstalk between biotic and abiotic stress responses involving ABA action. Studies reporting Ca^2+^ oscillations elicited by stimulating ABA and the temporal dynamics of Ca^2+^ in ABA signaling provide strong evidence showing that Ca^2+^ is a crucial element in the ABA signaling network [25]. The integration of ABA and calcium signalings could govern PP2C-type phosphatase regulators in the responses to abiotic stresses via the modulation of common targets [12]. Our comparative transcriptome profiles demonstrated that upregulated Ca^2+^-related genes in the *P1/HC-Pro^Tu^* plants were associated with ABA signaling (Figure 2 and Figure 3; and Table 4 and Table 5). The integration of both ABA and Ca^2+^ signaling processes might occur and simultaneously induce stress responses to P1/HC-Pro^Tu^ during exposure to drought/cold stress responses and particularly stomatal dynamics. Interestingly, an antagonistic regulatory mechanism controls stomatal movement via crosstalk among ABA, JA, and SA when pathogen effectors, i.e., P1/HC-Pro^Tu^, ingress into host tissues to induce a rapid defense response (Figure 5A). In addition, the *P1/HC-Pro^Tu^* plants exhibited upregulated genes associated with SA and brassinosteroid (BR) signaling, including *NPR3* (AT5G45110), which is involved in the negative regulation of defense responses against bacteria [26], and *BAR1* (AT5G18360) and *BKI1* (AT5G42750), which have Ca^2+^-dependent functions [27]. These results provided possible links among ABA, other phytohormones, and the secondary messenger calcium in stimulus-response reactions of the *P1/HC-Pro^Tu^* plants.

### 4.3. The LTP NGS Strategy Enables the Collection of a Miniature of the HTP Sequencing Data

RNA silencing in plants prevents virus accumulation [28,29], and accordingly, viruses have evolved various strategies to counteract this defense. Viral silencing of suppressor proteins blocks the production of siRNAs or the ability of siRNAs to reach their targets [30,31]. Previous studies proposed models for interfering with viral suppressors in endogenous silencing that contribute to viral symptom development [32,33]; however, the link between plant physiology and the underlying molecular mechanisms remains unclear. NGS technology enables an understanding of the roles of viral suppressors in stress responses and gene silencing mechanisms. Recently, a HTP transcriptome was applied in studies of the mechanism of virus-infected plant cells, and this approach enables a more accurate determination of plant-virus interactions [34,35]. Using a combination of microarray and RNA-Seq data, researchers can identify the molecular mechanisms and physiological alterations that might contribute to viral symptom development during acute infection [36]. Pasin et al. (2020) demonstrated that ABA can control RNA biology, including RNA stability, turnover, maturation, and translation, in viral suppressor transgenic plants [5].

Compared with the HTP NGS, LTP NGS represents a more economical approach for transcriptome studies due to the pooling of additional sample libraries. In this study, the DEGs identified using the LTP profiles could reflect the essence of the HTP profiles because we performed sequencing at only one-tenth of the sequencing depth used in the HTP analysis. The important genes correlated with drought stress, cold stress, senescence, and the RNA silencing pathway were accurately identified with the LTP dataset. Seventy-five network genes were identified with both the HTP and the LTP profiles, and these network genes included gene encoding PTGS components (i.e., *AGO1* and *AGO2*) and miRNA targets (i.e., *SPL3*, *SPL13A*; *CSD1*, *CSD2*, *CCS*, and *TOE2*). These results imply that the LTP strategy has a certain level of accuracy with respect to transcriptome analysis. However, the Pearson correlation coefficients were not 100% identical between the HTP and LTP profiles. A low number of genes might be excluded in the LTP analysis, which would lead to differences in matrix genes and thus differences in the Pearson correlation coefficients between the HTP and LTP profiles. Therefore, we recommend that the LTP approach can be used in preliminary or primary studies to identify critical genes and to identify a backbone network before a more in-depth HTP approach is considered.

## 5. Conclusions

By using comparative networks from the HTP RNA-Seq profiles, it revealed that P1/HC-Pro^Tu^ can trigger multiple phytohormone-mediated stress responses, especially the regulatory signaling pathways controlled by ABA/Ca^2+^. Upon stimulation by P1/HC-Pro^Tu^, expression changes of genes involved in various cellular processes, such as drought and cold stress responses, stomatal dynamics, senescence, cell death, and chlorophyll degradation, were linked to viral suppressor induced responses. The P1/HC-Pro^Tu^-mediated ABA disturbances via PTGS suppression might alter these gene expressions. Using a LTP NGS approach, we can simulate the HTP network for studying the P1/HC-Pro^Tu^-mediated PTGS suppression.

## Figures and Tables

**Table 1:** Identification of the HTP and LTP network genes in the Col-0 vs. *P1/HC-Pro^Tu^*, Col-0 vs. *P1^Tu^*, and Col-0 vs. *HC-Pro^Tu^* comparative datasets in the ContigViews system.

	HTP			LTP		
	Col-0 vs. *P1/HC-Pro^Tu^*	Col-0 vs. *P1^Tu^*	Col-0 vs. *HC-Pro^Tu^*	Col-0 vs. *P1/HC-Pro^Tu^*	Col-0 vs. *P1^Tu^*	Col-0 vs. *HC-Pro^Tu^*
Passing rate	80	80	80	80	80	80
Fold change	2	2	2	2	2	2
(+) correlation	97.5	97.5	97.5	95	95	95
(−) correlation	92.5	92.5	92.5	90	90	90
Sample sets	10/12	10/12	10/12	10/12	10/12	10/12
Filtered DEGs	1601	559	777	700	621	587
Network genes	662	106	162	188	243	193

**Table 2:** List of genes involved in the ABA signaling pathway that were identified in the *P1/HC-Pro^T^^u^*-only section of the HTP profiles.

Categories	Subcategories	AGI Locus Code	Gene Name	Annotations
Homeostasis	Biosynthesis	AT3G02570	*PMI1/MEE31*	Mannose-6-phosphate isomerase, type I
		AT3G51895	*SULTR3;1*	sulfate transporter 3;1
	Catabolism	AT5G45340	*CYP707A3*	Cytochrome P450, family 707, subfamily A, polypeptide 3
	Transport	AT5G52050	*DTX50*	MATE efflux family protein
Regulation	Posttranscriptional regulation	AT5G66070	*ATL27/ATARRE*	RING/U-box superfamily protein
		AT3G52450	*PUB22*	Plant U-box 22
		AT2G35930	*PUB23*	Plant U-box 23
		AT3G46620	*RDUF1*	Zinc finger (C3HC4-type RING finger) family protein
		AT5G59550	*RDUF2/BTL09*	Zinc finger (C3HC4-type RING finger) family protein
	Transcriptional regulation	AT3G61890	*HB12*	Homeobox 12
ABA-responses	Biotic	AT1G69480	*EXS*	EXS (ERD1/XPR1/SYG1) family protein
		AT1G32640	*MYC2*	Basic helix-loop-helix (bHLH) DNA-binding family protein
		AT2G32030	NA ^a^	Acyl-CoA N-acyltransferases (NAT) superfamily protein
		AT2G19810	*OZF1/TZF2*	CCCH-type zinc finger family protein
	Cold	AT4G25470	*CBF2*	C-repeat/DRE binding factor 2
	Cold	AT5G61600	*ERF104*	Ethylene response factor 104
	Cold	AT5G51190	*ERF105*	Integrase-type DNA-binding superfamily protein
	Cold, Development	AT1G74670	*GASA6*	Gibberellin-regulated family protein
	Development	AT1G56170	*NF-YC2/HAP5B*	Nuclear factor Y, subunit C2
	Development	AT5G23220	*NIC3*	Nicotinamidase 3
	Development	AT5G42050	*NRP*	DCD (Development and Cell Death) domain protein, NRP has a positive role in ABA-mediated seed germination.
	Development, Drought	AT1G13260	*RAV1*	Related to ABI3/VP1 1
	Development, Drought	AT5G39760	*HB23*	Homeobox protein 23
	Drought	AT2G46510	*AIB/BHLH017*	ABA-inducible BHLH-type transcription factor
	Drought	AT2G25900	*ATCTH/TZF1*	Zinc finger C-x8-C-x5-C-x3-H type family protein
	Drought	AT1G74930	*ERF018/ORA47*	Integrase-type DNA-binding superfamily protein
	Drought	AT5G62470	*MYB96*	MYB domain protein 96
	Drought	AT5G67300	*MYBR1/MYB44*	MYB domain protein r1
	Drought	AT3G56880	NA ^a^	VQ motif-containing protein
	Drought	AT1G54160	*NF-YA5*	Nuclear factor Y, subunit A5
	Drought	AT2G39800	*P5CS1*	Delta1-pyrroline-5-carboxylate synthase 1
	Drought	AT3G11820	*SYR1/SYP121*	Syntaxin of plants 121
	Drought	AT2G46400	*WRKY46*	WRKY DNA-binding protein 46
	Drought, Cold	AT5G65300	*SUPA*	Unknown protein
	Drought, Cold	AT1G72240	*SUPA-like*	Unknown protein
	Drought, Senescence	AT1G08920	*ESL1*	ERD (early response to dehydration) six-like 1
	Drought, Senescence, Chlorophyll degradation	AT1G69490	*ANAC029/NAP*	NAC-like, activated by AP3/PI
	Multiple stress	AT3G48360	*BT2*	BTB and TAZ domain protein 2
	Senescence	AT1G53170	*ERF8*	Ethylene response factor 8
	Senescence, Chlorophyll degradation	AT4G24390	*AFB4*	RNI-like superfamily protein
	Uncertain	AT3G54200	*NHL39*	Late embryogenesis abundant (LEA) hydroxyproline-rich glycoprotein family

^a^ NA indicates that the gene name is not available.

**Table 3:** List of genes in the calcium signaling pathway found in the *P1/HC-Pro^T^^u^*-only section of the HTP profiles.

Categories	Subcategories	AGI Locus Code	Gene Name	Annotations
Transporter	Uniporter	AT1G27770	*ACA1*	Autoinhibited Ca^2+^-ATPase 1
		AT2G24610	*CNGC14*	Cyclic nucleotide-gated channel 14
		AT5G15410	*CNGC2/DND1*	Cyclic nucleotide-regulated ion channel family protein
		AT5G17850	*CCX2*	Sodium/calcium exchanger family protein
		AT5G66650	*CMCU*	Protein of unknown function (DUF607)
Sensor	CaMs/CaMLs	AT1G66400	*CML23*	Calmodulin like 23
		AT1G76650	*CML38*	Calmodulin-like 38
		AT2G43290	*CML5/MSS3*	Calcium-binding EF-hand family protein
		AT3G22930	*CML11*	Calmodulin-like 11
		AT5G37770	*CML24/TCH2*	EF hand calcium-binding protein family
		AT5G39670	*CML46*	Calcium-binding EF-hand family protein
	Other calcium-binding proteins	AT3G01830	NA ^a^	Calcium-binding EF-hand family protein
		AT3G16510	*CaLB*	Calcium-dependent lipid-binding (CaLB domain) family protein
		AT3G25600	NA ^a^	Calcium-binding EF-hand family protein
		AT4G34150	*CaLB*	Calcium-dependent lipid-binding (CaLB domain) family protein
	Calmodulin-binding proteins	AT2G26190	*IQM4*	Calmodulin-binding family protein
		AT2G41010	*CAMBP25*	Calmodulin (CAM)-binding protein of 25 kDa
		AT3G13600	NA ^a^	Calmodulin-binding family protein
		AT3G16490	*IQD26*	IQ-domain 26
		AT5G26920	*CBP60G*	Cam-binding protein 60-like G
		AT5G35670	*IQD33*	IQ-domain 33
		AT5G57010	NA ^a^	Calmodulin-binding family protein
		AT5G62570	*CBP60A*	Calmodulin binding protein-like
	CDPKs/CIPK	AT2G30360	*CIPK11/PKS5*	SOS3-interacting protein 4
		AT3G57530	*CPK32*	Calcium-dependent protein kinase 32
		AT5G66210	*CPK28*	Calcium-dependent protein kinase 28

^a^ NA indicates that the gene name is not available.

**Table 4:** List of genes related to drought responses found in the *P1/HC-Pro^Tu^*-only section of the HTP profiles.

AGI	Gene Name	Annotations	Categories ^b^	ABA/Ca ^c^
AT2G39800	*P5CS1*	Delta1-pyrroline-5-carboxylate synthase 1	drought/stomata	ABA
AT3G46620	*RDUF1*	Zinc finger (C3HC4-type RING finger) family protein	drought/stomata	ABA
AT5G59550	*RDUF2/BTL09*	Zinc finger (C3HC4-type RING finger) family protein	drought/stomata	ABA
AT3G11820	*SYR1/SYP121*	Syntaxin of plants 121	drought/stomata	ABA
AT2G46400	*WRKY46*	WRKY DNA-binding protein 46	drought/stomata	ABA
AT1G32640	*MYC2*	Basic helix-loop-helix (bHLH) DNA-binding family protein	drought/stomata	ABA
AT3G57230	*AGL16*	AGAMOUS-like 16	drought/stomata	
AT3G23920	*BAM1*	Beta-amylase 1	drought/stomata	
AT1G34245	*EPF2*	Putative membrane lipoprotein	drought/stomata	
AT5G10720	*HK5*	Histidine kinase 5	drought/stomata	
AT5G22920	*RZPF34/CHYR1*	CHY-type/CTCHY-type/RING-type Zinc finger protein	drought/stomata	
AT3G52400	*SYP122*	Syntaxin of plants 122	drought/stomata	
AT1G74950	*TIFY10B/JAZ2*	TIFY domain/Divergent CCT motif family protein	drought/stomata	
AT1G80080	*TMM*	Leucine-rich repeat (LRR) family protein	drought/stomata	
AT1G52890	*ANAC019/NAC019*	NAC domain containing protein 19	drought/stomata	
AT1G69490	*ANAC029/NAP*	NAC-like, activated by AP3/PI	drought	ABA
AT2G25900	*ATCTH/TZF1*	Zinc finger C-x8-C-x5-C-x3-H type family protein	drought	ABA
AT5G45340	*CYP707A3*	Cytochrome P450, family 707, subfamily A, polypeptide 3	drought	ABA
AT5G52050	*DTX50*	MATE efflux family protein	drought	ABA
AT1G74930	*ERF018/ORA47*	Integrase-type DNA-binding superfamily protein	drought	ABA
AT1G08920	*ESL1*	ERD (early response to dehydration) six-like 1	drought	ABA
AT5G39760	*HB23*	Homeobox protein 23	drought	ABA
AT5G62470	*MYB96*	MYB domain protein 96	drought	ABA
AT5G67300	*MYBR1/MYB44*	MYB domain protein r1	drought	ABA
AT1G54160	*NF-YA5*	Nuclear factor Y, subunit A5	drought	ABA
AT5G42050	*NRP*	DCD (Development and Cell Death) domain protein	drought	ABA
AT3G52450	*PUB22*	Plant U-box 22	drought	ABA
AT2G35930	*PUB23*	Plant U-box 23	drought	ABA
AT1G13260	*RAV1*	Related to ABI3/VP1 1	drought	ABA
AT5G65300	*SUPA*	Unknown protein	drought	ABA
AT2G41010	*CAMBP25*	Calmodulin (CAM)-binding protein of 25 kDa	drought	Ca
AT2G26190	*IQM4*	Calmodulin-binding family protein	drought	Ca
AT5G17850	*CCX2*	Sodium/calcium exchanger family protein	drought	Ca
AT5G66650	*CMCU*	Protein of unknown function (DUF607)	drought	Ca
AT2G33860	*ARF3/ETT*	Transcriptional factor B3 family protein/auxin-responsive factor AUX/IAA-related	drought	
AT4G02200	*AtDi19-5*	Drought-responsive family protein	drought	
AT3G08760	*ATSIK*	Protein kinase superfamily protein	drought	
AT5G49450	*BZIP1*	Basic leucine-zipper 1	drought	
AT4G36880	*CP1/RDL1*	Cysteine proteinase1	drought	
AT5G04340	*CZF2/ZAT6*	Zinc finger of Arabidopsis thaliana 6	drought	
AT5G04760	*DIV2*	Duplicated homeodomain-like superfamily protein	drought	
AT1G73330	*DR4*	Drought-repressed 4	drought	
AT5G05410	*DREB2A*	DRE-binding protein 2A	drought	
AT2G45180	*DRN1*	Bifunctional inhibitor/lipid-transfer protein/seed storage 2S albumin superfamily protein	drought	
AT4G17490	*ERF6*	Ethylene responsive element binding factor 6	drought	
AT2G47180	*GOLS1*	Galactinol synthase 1	drought	
AT4G18880	*HSF A4A*	Heat shock transcription factor A4A	drought	
AT5G12030	*HSP17.6A*	Heat shock protein 17.6A	drought	
AT4G02410	*LPK1/LECRK-IV.3*	Concanavalin A-like lectin protein kinase family protein	drought	
AT3G49580	*LSU1*	Response to low sulfur 1	drought	
AT1G73500	*MKK9*	MAP kinase kinase 9	drought	
AT3G45640	*MPK3*	Mitogen-activated protein kinase 3	drought	
AT5G61290	NA ^a^	Flavin-binding monooxygenase family protein	drought	
AT3G27150	NA ^a^	Galactose oxidase/kelch repeat superfamily protein	drought	
AT5G26260	NA ^a^	TRAF-like family protein	drought	
AT4G33070	*PDC1*	Thiamine pyrophosphate dependent pyruvate decarboxylase family protein	drought	
AT3G62260	*PP2C49*	Protein phosphatase 2C family protein	drought	
AT5G64905	*PROPEP3*	Elicitor peptide 3 precursor	drought	
AT3G18710	*PUB29*	plant U-box 29	drought	
AT3G49810	*PUB30*	ARM repeat superfamily protein	drought	
AT1G68840	*RAV2/TEM2*	Related to ABI3/VP1 2	drought	
AT3G08720	*S6K2*	Serine/threonine protein kinase 2	drought	
AT5G62520	*SRO5*	Similar to RCD one 5	drought	
AT3G55980	*SZF1/TZF11*	Salt-inducible zinc finger 1	drought	
AT2G40140	*SZF2*	Zinc finger (CCCH-type) family protein	drought	
AT1G27730	*ZAT10*	Salt tolerance zinc finger	drought	

^a^ NA indicates that the gene name is not available. ^b^ Classification of the genes into the functional categories of drought responses (drought, salt, osmotic, salinity, dehydration, water loss, submergence, and water loading) and/or stomatal dynamics. ^c^ Involvement of the genes in the ABA and Ca^2+^ signaling pathways.

**Table 5:** List of genes related to the cold response found in the *P1/HC-Pro^Tu^*-only section of the HTP profiles.

AGI	Gene Name	Annotations	Categories ^a^	ABA/Ca ^b^
AT5G61600	*ERF104*	Ethylene response factor 104	cold	ABA
AT5G51190	*ERF105*	Integrase-type DNA-binding superfamily protein	cold	ABA
AT1G13260	*RAV1*	Related to ABI3/VP1 1	cold/drought	ABA
AT4G25470	*CBF2*	C-repeat/DRE binding factor 2	cold	ABA/Ca
AT3G50260	*CEJ1/DEAR1*	Cooperatively regulated by ethylene and jasmonate 1	cold	Ca
AT5G59820	*ZAT12*	C2H2-type zinc finger family protein	cold	Ca
AT1G27730	*ZAT10*	Salt tolerance zinc finger	cold/drought	Ca
AT5G05410	*DREB2A*	DRE-binding protein 2A	cold/drought	
AT4G17490	*ERF6*	Ethylene responsive element binding factor 6	cold/drought	
AT2G47180	*GOLS1*	Galactinol synthase 1	cold/drought	
AT3G45640	*MPK3*	Mitogen-activated protein kinase 3	cold/drought	
AT3G08720	*S6K2*	Serine/threonine protein kinase 2	cold/drought	
AT1G20823	*ATL80*	RING/U-box superfamily protein	cold	
AT1G18740	*B1L*	Protein of unknown function (DUF793)	cold	
AT5G20230	*BCB*	Blue-copper-binding protein	cold	
AT3G13310	*DJC66*	Chaperone DnaJ-domain superfamily protein	cold	
AT3G49530	*NAC062/NTL6*	NAC domain containing protein 62	cold	
AT1G09070	*SRC2*	Soybean gene regulated by cold-2	cold	
AT1G72940	*TN11*	Toll-Interleukin-Resistance (TIR) domain-containing protein	cold	
AT5G57560	*XTH22/TCH4*	Xyloglucan endotransglucosylase/hydrolase family protein	cold	

^a^ Classification of the genes into the functional categories of cold responses (cold, low temperature, freeze, and chilling) and/or drought. ^b^ Involvement of the genes in the ABA and/or calcium signaling pathways.

**Table 6:** List of genes related to senescence, cell death, and chlorophyll degradation found in the *P1/HC-Pro^Tu^*-only section of the HTP datasets.

AGI	Gene Name	Annotations	Categories ^b^
AT5G42050	*NRP*	DCD (Development and Cell Death) domain protein	cell death
AT3G25250	*AGC2-1/OXI1*	AGC (cAMP-dependent, cGMP-dependent and protein kinase C) kinase family protein	cell death
AT5G18270	*ANAC087*	Arabidopsis NAC domain containing protein 87	cell death
AT1G29690	*CAD1/NSL2*	MAC/Perforin domain-containing protein	cell death
AT5G58430	*EXO70B1*	Exocyst subunit exo70 family protein B1	cell death
AT1G59910	*FORMIN7*	Actin-binding FH2 (formin homology 2) family protein	cell death
AT1G70830	*MLP28*	MLP-like protein 28	cell death
AT4G12720	*NUDT7*	MutT/nudix family protein	cell death
AT1G08920	*ESL1*	ERD (early response to dehydration) six-like 1	senescence
AT2G46400	*WRKY46*	WRKY DNA-binding protein 46	senescence
AT5G02760	*APD7*	Protein phosphatase 2C family protein	senescence
AT5G08790	*ATAF2/ANAC081*	NAC (No Apical Meristem) domain transcriptional regulator superfamily protein	senescence
AT1G44830	*ERF014/EPI1*	Integrase-type DNA-binding superfamily protein	senescence
AT1G51660	*MKK4*	Mitogen-activated protein kinase kinase 4	senescence
AT1G67470	NA ^a^	Protein kinase superfamily protein	senescence
AT2G28400	NA ^a^	Protein of unknown function, DUF584	senescence
AT4G21930	NA ^a^	Protein of unknown function, DUF584	senescence
AT2G44670	NA ^a^	Protein of unknown function, DUF581	senescence
AT4G04630	NA ^a^	Protein of unknown function, DUF584	senescence
AT1G55740	*SIP1*	Seed imbibition 1	senescence
AT4G01250	*WRKY22*	WRKY family transcription factor	senescence
AT3G21520	*DMP1*	DUF679 domain membrane protein 1	senescence/cell death
AT1G52890	*ANAC019/NAC019*	NAC domain containing protein 19	senescence/chloroplast degradation
AT1G69490	*ANAC029/NAP*	NAC-like, activated by AP3/PI	senescence/chloroplast degradation
AT4G24390	*AFB4*	RNI-like superfamily protein	senescence/chloroplast degradation

^a^ NA indicates that the gene name is not available. ^b^ Classification of the gene into the functional categories of senescence responses (leaf senescence, chloroplast degradation, and cell death).

**Table 7:** Statistics of the RNA-seq data and read mapping rates of the Col-0, *P1^Tu^*, *HC-Pro^Tu^*, and *P1/HC-Pro^Tu^*libraries obtained with the HTP and LTP profiles.

Samples ^a^		Read Length (bp)	Raw Reads	Clear Reads	Mapped Rates (% of Total)
HTP	Col-0-1	125	23,692,678	23,692,678	82.09
	Col-0-2	125	23,536,690	23,536,690	81.41
	Col-0-3	125	24,361,660	24,361,660	84.04
	*P1* *^Tu^-1*	125	24,270,634	24,270,634	79.83
	*P1* *^Tu^-2*	125	23,727,160	23,727,160	79.97
	*P1* *^Tu^-3*	125	24,663,280	24,663,280	79.54
	*HC-Pro* *^Tu^-1*	125	23,502,832	23,502,832	77.45
	*HC-Pro* *^Tu^-2*	125	23,074,688	23,074,688	80.87
	*HC-Pro* *^Tu^-3*	125	23,817,246	23,817,246	79.23
	*P1/HC-Pro* *^Tu^-1*	125	23,104,480	23,104,480	75.27
	*P1/HC-Pro* *^Tu^-2*	125	23,766,692	23,766,692	73.56
	*P1/HC-Pro* *^Tu^-3*	125	21,935,512	21,935,512	77.24
LTP	Col-0-1	75	2,732,672	2,611,636	78.17
	Col-0-2	75	3,027,192	2,895,782	78.97
	Col-0-3	75	2,974,324	2,844,916	79.84
	*P1* *^Tu^-1*	75	3,072,748	2,899,822	80.16
	*P1* *^Tu^-2*	75	3,100,226	2,956,540	79.33
	*P1* *^Tu^-3*	75	2,309,898	2,151,492	80.63
	*HC-Pro* *^Tu^-1*	75	2,300,776	2,196,490	77.28
	*HC-Pro* *^Tu^-2*	75	2,814,492	2,664,142	78.34
	*HC-Pro* *^Tu^-3*	75	1,967,520	1,888,408	77.94
	*P1/HC-Pro* *^Tu^-1*	75	2,738,024	2,609,924	76.81
	*P1/HC-Pro* *^Tu^-2*	75	3,012,322	2,866,212	77.07
	*P1/HC-Pro* *^Tu^-3*	75	2,973,652	2,817,066	77.53

^a^ 1, 2, and 3 represent three independent biological replicates.

**Table 8:** List of 75 overlapping genes in the *P1/HC-Pro^Tu^*-only section identified from both the HTP and LTP profiles.

AGI	Gene Name	Annotation	Functional Classification ^b^	ABA/Ca ^c^	Stresses ^d^
AT1G48410	*AGO1*	Stabilizer of iron transporter SufD/Polynucleotidyl transferase	▇		
AT1G31280	*AGO2*	Argonaute family protein	▇		
AT4G12720	*NUDT7*	MutT/nudix family protein	▇		cell death
AT3G49530	*NAC062/NTL6*	NAC domain containing protein 62	▇		cold
AT5G57560	*XTH22/TCH4*	Xyloglucan endotransglucosylase/hydrolase family protein	▇		cold
AT5G67300	*MYBR1/MYB44*	MYB domain protein r1	▇	ABA	drought
AT3G08760	*ATSIK*	Protein kinase superfamily protein	▇		drought
AT1G73330	*DR4*	Drought-repressed 4	▇		drought
AT4G18880	*HSF A4A*	Heat shock transcription factor A4A	▇		drought
AT3G49810	*PUB30*	ARM repeat superfamily protein	▇		drought
AT3G08720	*S6K2*	Serine/threonine protein kinase 2	▇		drought, cold
AT1G51660	*MKK4*	mitogen-activated protein kinase kinase 4	▇		senescence
AT4G01250	*WRKY22*	WRKY family transcription factor	▇		senescence
AT3G48360	*BT2*	BTB and TAZ domain protein 2	▇	ABA	
AT1G33560	*ADR1*	Disease resistance protein (CC-NBS-LRR class) family	▇		
AT1G08830	*CSD1*	Copper/zinc superoxide dismutase 1	▇		
AT2G28190	*CSD2*	Copper/zinc superoxide dismutase 2	▇		
AT5G41750	*DM1*	Disease resistance protein (TIR-NBS-LRR class) family	▇		
AT5G41080	*GDPD2*	PLC-like phosphodiesterases superfamily protein	▇		
AT1G25550	*HHO3/NIGT1.1*	MYB-like transcription factor family protein	▇		
AT3G10020	*HUP26*	Unknown protein; response to oxidative stress, anaerobic respiration	▇		
AT1G17420	*LOX3*	Lipoxygenase 3	▇		
AT1G72520	*LOX4*	PLAT/LH2 domain-containing lipoxygenase family protein	▇		
AT2G01180	*LPP1*	Phosphatidic acid phosphatase 1	▇		
AT1G01560	*MPK11*	MAP kinase 11	▇		
AT4G37260	*MYB73*	MYB domain protein 73	▇		
AT1G66090	*TIR-NBS-LRR*	Disease resistance protein (TIR-NBS class)	▇		
AT5G04720	*PHX21/ADR1-L2*	ADR1-like 2	▇		
AT4G17230	*SCL13*	SCARECROW-like 13	▇		
AT1G19020	*SDA1/HUP35*	Unknown protein	▇		
AT2G23810	*TET8*	Tetraspanin8	▇		
AT2G33770	*UBC24/PHO2*	Phosphate 2	▇		
AT1G56250	*VBF/PP2-B14*	Phloem protein 2-B14	▇		
AT1G12520	*CCS*	Copper chaperone for SOD1	▇		
AT1G70830	*MLP28*	MLP-like protein 28	▇		cell death
AT5G02760	*APD7*	Protein phosphatase 2C family protein	▇		senescence
AT1G74670	*GASA6*	Gibberellin-regulated family protein	▇	ABA	
AT5G27420	*ATL31/CNI1*	Carbon/nitrogen insensitive 1	▇		
AT2G22840	*GRF1*	Growth-regulating factor 1	▇		
AT3G05690	*NF-YA2/HAP2B*	Nuclear factor Y, subunit A2	▇		
AT3G14020	*NF-YA6*	Nuclear factor Y, subunit A6	▇		
AT5G50570	*SPL13A*	Squamosa promoter-binding protein-like (SBP domain) transcription factor family protein	▇		
AT2G33810	*SPL3*	Squamosa promoter binding protein-like 3	▇		
AT5G60120	*TOE2*	Target of early activation tagged (EAT) 2	▇		
AT1G69530	*EXPA1*	Expansin A1	▇		
AT2G26190	*IQM4*	Calmodulin-binding family protein	▇	Ca	drought
AT4G34150	*CaLB*	Calcium-dependent lipid-binding (CaLB domain) family protein	▇	Ca	
AT3G16510	*CaLB*	Calcium-dependent lipid-binding (CaLB domain) family protein	▇	Ca	
AT5G26920	*CBP60G*	Cam-binding protein 60-like G	▇	Ca	
AT5G37770	*CML24/TCH2*	EF hand calcium-binding protein family	▇	Ca	
AT5G66210	*CPK28*	Calcium-dependent protein kinase 28	▇	Ca	
AT3G57530	*CPK32*	Calcium-dependent protein kinase 32	▇	Ca	
AT1G27770	*ACA1*	Autoinhibited Ca^2+^-ATPase 1	▇	Ca	
AT5G45340	*CYP707A3*	Cytochrome P450, family 707, subfamily A, polypeptide 3	▇	ABA	drought
AT5G08790	*ATAF2/ANAC081*	NAC (No Apical Meristem) domain Transcriptional regulator superfamily protein	▇		senescence
AT5G66070	*ATL27/ATARRE*	RING/U-box superfamily protein	▇	ABA	
AT3G25780	*AOC3*	Allene oxide cyclase 3	▇		
AT1G28370	*ERF11*	ERF domain protein 11	▇		
AT4G36040	*J11*	Chaperone DnaJ-domain superfamily protein	▇		
AT4G23180	*CRK10/RLK4*	Cysteine-rich RLK (RECEPTOR-like protein kinase) 10			
AT3G26200	*CYP71B22*	Cytochrome P450, family 71, subfamily B, polypeptide 22			
AT1G80610	NA^a^	Unknown protein			
AT1G63750	*TIR-NBS-LRR*	Disease resistance protein (TIR-NBS-LRR class) family			
AT4G32480	NA ^a^	Protein of unknown function (DUF506)			
AT3G15450	NA ^a^	Aluminium induced protein with YGL and LRDR motifs			
AT4G23870	NA ^a^	Unknown protein			
AT5G46780	NA ^a^	VQ motif-containing protein			
AT2G39650	NA ^a^	Protein of unknown function (DUF506)			
AT1G77270	NA ^a^	Unknown protein			
AT2G29300	NA ^a^	NAD(P)-binding Rossmann-fold superfamily protein			
AT3G07350	NA^a^	Protein of unknown function (DUF506)			
AT1G52820	NA ^a^	2-oxoglutarate (2OG) and Fe(II)-dependent oxygenase superfamily protein			
AT3G44400	NA ^a^	Disease resistance protein (TIR-NBS-LRR class) family			
AT5G44572	*PROSCOOP6*	Unknown protein			
AT1G16110	*WAKL6*	Wall associated kinase-like 6			

^a^ NA indicates that the gene name is not available. ^b^ Functional classification of genes based on the color shown in the corresponding network. ^c^ Involvement of genes in the ABA and Ca^2+^ signaling pathways. ^d^ Classification of genes into the functional categories of drought responses (drought, salt, osmotic, salinity, dehydration, water loss, submergence, and water loading), cold responses (cold, low temperature, freeze, and chilling) and senescence responses (leaf senescence, chloroplast degradation, and cell death).

## Data Availability

The data presented in this study are available on request from the corresponding author.
